# OTUB1 Modulates Ferroptosis by Regulating SLC7A11 Ubiquitination in Pancreatic β‐Cells

**DOI:** 10.1096/fj.202502289R

**Published:** 2025-10-12

**Authors:** Soyeon Yoo, Dongkyu Kim, Miyeon Kim, Ju Young Bae, Sang Ah Lee, Eui Tae Kim, Gwanpyo Koh

**Affiliations:** ^1^ Department of Internal Medicine Jeju National University College of Medicine Jeju Republic of Korea; ^2^ Department of Internal Medicine Jeju National University Hospital Jeju Republic of Korea; ^3^ Department of Microbiology & Immunology Jeju National University College of Medicine Jeju Republic of Korea; ^4^ Jeju Research Center for Natural Medicine Jeju National University Core Research Institute Jeju Republic of Korea

**Keywords:** 2‐deoxy‐d‐ribose, ferroptosis, OTUB1, SLC7A11, β‐cell

## Abstract

Ferroptosis, a regulated form of cell death driven by iron‐dependent lipid peroxidation, plays a significant role in pancreatic β‐cell failure associated with diabetes. In this study, we investigated how 2‐deoxy‐d‐ribose (dRib) induces ferroptosis in β‐cells and identified the OTUB1–SLC7A11 axis as a critical regulatory mechanism. Using rat insulinoma‐derived RIN5mF cells and isolated rat islets, we found that dRib exposure markedly impaired cystine uptake via system χc^−^, resulting in intracellular glutathione (GSH) depletion, accumulation of lipid peroxidation products such as malondialdehyde and 4‐hydroxynonenal, and increased lipid reactive oxygen species (ROS). Although SLC7A11 mRNA levels were upregulated, its protein levels were reduced due to increased ubiquitination and proteasomal degradation. Further analysis revealed that dRib suppressed the expression of OTUB1, a deubiquitinating enzyme that directly interacts with SLC7A11 and inhibits its ubiquitination. Overexpression of either OTUB1 or SLC7A11 restored cystine transport, replenished GSH levels, reduced lipid ROS and cell death, supporting their protective roles against ferroptosis. Transmission electron microscopy confirmed that dRib induced mitochondrial morphological changes consistent with ferroptosis, such as shrinkage and cristae loss. These findings demonstrate that the loss of OTUB1 promotes ferroptosis in pancreatic β‐cells through destabilization of SLC7A11. Targeting the OTUB1–SLC7A11 pathway may offer a novel therapeutic approach for preserving β‐cell survival and preventing the progression of diabetes.

## Graphical abstract

2‐Deoxy‐d‐ribose (dRib) suppresses OTUB1 expression in pancreatic β‐cells, reducing its ability to stabilize SLC7A11 (xCT). Loss of OTUB1 enhances SLC7A11 ubiquitination and proteasomal degradation, leading to impaired cystine uptake, glutathione depletion, and ferroptotic cell death. Conversely, OTUB1 normally prevents SLC7A11 degradation and maintains redox balance. These findings highlight the OTUB1–SLC7A11 axis as a novel regulatory pathway of β‐cell ferroptosis and a potential therapeutic target in diabetes.

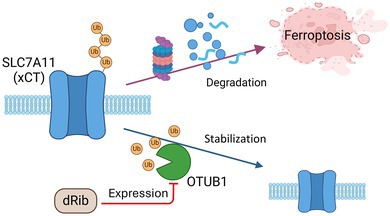

## 1 Introduction

Cell death is broadly categorized into accidental cell death (necrosis) and regulated cell death, including apoptosis and regulated necrosis [1]. Ferroptosis, a form of regulated necrosis, is characterized by iron‐dependent lipid peroxidation initiated by reactive oxygen species (ROS) through the Fenton reaction [2]. This process involves a chain reaction in which phospholipid radicals generated from polyunsaturated fatty acids (PUFAs) propagate membrane damage [3, 4]. Ferroptosis is biochemically distinct from other cell death types and has emerged as a key mechanism in various diseases, including cancer, neurodegeneration, and kidney disease [5].

Iron overload is a known risk factor for diabetes and is implicated in both hereditary hemochromatosis and transfusion‐related diabetes [6, 7]. Clinical evidence suggests that iron reduction therapies, such as phlebotomy, improve glycemic control [8]. Pancreatic β‐cells are particularly vulnerable to oxidative damage due to high iron content [9] and low antioxidant capacity [10], making them susceptible to ferroptosis [11]. Furthermore, glucose toxicity, which is considered to be a mechanism of β‐cell failure in type 2 diabetes, increases the iron content in β‐cells and decreases their insulin secretory capacity [12, 13].

In previous work, we found that 2‐deoxy‐d‐ribose (dRib), a reducing sugar used to model glucotoxicity, causes oxidative damage in pancreatic β‐cells by disrupting intracellular cystine transport via system χc^−^ (14). We also reported that in renal tubular epithelial cells (RTECs), dRib induces ferroptosis by increasing the ubiquitination and degradation of SLC7A11, the light‐chain subunit of system χc^−^ (15). Therefore, in the present study, we aimed to determine whether dRib also induces ferroptosis in pancreatic β‐cells and to elucidate its underlying mechanism. Unlike previous studies that focused on classical ferroptosis triggers, we investigated a novel mechanism in which dRib promotes ferroptosis by reducing OTUB1 expression, leading to increased ubiquitination and degradation of SLC7A11. OTUB1 has not previously been studied in the context of β‐cell ferroptosis. To our knowledge, this is the first study to demonstrate that the OTUB1‐SLC7A11 axis is a key regulatory pathway for ferroptosis in pancreatic β‐cells. Furthermore, we explored whether overexpression of OTUB1 or SLC7A11 could mitigate dRib‐induced lipid peroxidation and β‐cell death. These findings suggest a new molecular target for protecting β‐cells from ferroptosis and advancing diabetes therapy.

## 2 Materials and Methods

### 2.1 Materials

dRib, NaCl, NaHCO_3_, KCl, MgSO_4_, K_2_HPO_4_, and HEPES were obtained from Amresco. RPMI‐1640 medium, penicillin, streptomycin, trypsin–EDTA, Dulbecco's phosphate‐buffered saline (DPBS), and Hanks' balanced salt solution (HBSS) were acquired from Gibco Invitrogen. Fetal bovine serum (FBS) was purchased from HyClone. Radioactive l‐[^14^C]cystine and the scintillation cocktail were provided by PerkinElmer. Polyacrylamide gels and nitrocellulose membranes were sourced from Amersham. The acrylamide/bis‐acrylamide solution (30%) and bovine serum albumin (BSA) were obtained from Biosesang (South Korea) and Bovogen Biologicals (Australia), respectively. Other chemicals, including 2‐mercaptoethanol (2‐ME), CaCl_2_, d‐glucose, dimethyl sulfoxide, H_2_O_2_, polybrene, Triton‐X 100, DNase, collagenase P, Ficoll, deferoxamine (DFO), FeSO_4_, ferrostatin‐1 (Fer‐1), liproxstatin‐1 (Lip‐1), DNase I, collagenase type I, polybrene, and puromycin were purchased from Sigma‐Aldrich. All cell culture dishes and tubes were obtained from BD Biosciences.

### 2.2 Cell Culture and Islet Isolation

RIN5mF cells are a subclone of rat insulinoma cells that retain key characteristics of pancreatic β‐cells, making them a suitable model for studying β‐cell death [14]. Therefore, we selected RIN5mF cells as the cell line for our study and purchased them from the Korean Cell Line Bank (Seoul, Korea). Cells were cultured in RPMI‐1640 medium supplemented with 10% FBS, 100 mg/mL penicillin, and 100 mg/mL streptomycin. The cell cultures were maintained at 37°C in an atmosphere of 5% CO_2_ and 95% O_2_. The culture medium was replenished every 2 days, and cells were subcultured using trypsin when they reached approximately 70% confluence.

For islet isolation, Sprague Dawley rats (6–8 weeks old) were obtained from OrientBio Corp. (South Korea). Islet isolation and culture were performed as previously described [15]. All Sprague Dawley rats were euthanized using carbon dioxide (CO_2_) inhalation in accordance with the protocol approved by the Institutional Animal Care and Use Committee of Jeju National University (Protocol No. 2024‐0088). CO_2_ was administered in a gradually increasing concentration to minimize distress. Death was confirmed by the cessation of respiration and heartbeat, and the lack of response to physical stimuli. Following euthanasia, the islets were promptly isolated. This animal study protocol was approved by the Institutional Animal Care and Use Committee of Jeju National University (2022‐0014).

### 2.3 Measurement of Intracellular Cystine Transport

We measured the intracellular transport of l‐[^14^C]cystine in RIN5mF cells and islets using a slightly modified version of the method described by Tomi et al. [16]. The detailed method has been described previously [15, 17].

### 2.4 Measurement of Intracellular GSH Levels

We measured the total intracellular GSH content of RIN5mF cells and islets using a GSH assay kit (Cayman) based on the principle of enzymatic recycling with glutathione reductase.

### 2.5 Measurement of Intracellular Iron Levels

We measured intracellular total iron levels of RIN5mF cells using a colorimetric iron assay kit (Sigma‐Aldrich).

### 2.6 Assessment of Cell Viability

Cytotoxicity was evaluated using a lactate dehydrogenase (LDH) assay. RIN5mF cells (1 × 10^5^ cells/well) or isolated islets were seeded in 96‐well plates and cultured in RPMI‐1640 medium with 10% FBS. The cells were stimulated with 30 mM dRib and 100 μM DFO, with or without 100 μM FeSO_4_, 20 μM Fer‐1, or 20 μM Lip‐1 for 24 h. LDH release was measured using the Cytotoxicity Detection KitPLUS (Roche). Cytotoxicity was calculated and expressed as the percentage of viable cells (% of control).

### 2.7 Stable Cell Lines

Lentiviral particles encoding rat SLC7A11, OTUB1, or an empty vector were obtained from OriGene. RIN5mF cells were infected with lentiviral particles at a multiplicity of infection of 10 in the presence of 8 μg/mL polybrene and were cultured in 24‐well plates. After 1 week of selection using 1.4 μg/mL puromycin, stable cell lines expressing SLC7A11 or OTUB1 were established.

### 2.8 Measurement of Intracellular Malondialdehyde and 4‐Hydroxynonenal Levels

Malondialdehyde (MDA) and 4‐hydroxynonenal (4‐HNE) were quantified as markers of lipid peroxidation. RIN5mF cells (1 × 10^6^ cells/well) were cultured in six‐well plates and stimulated with 30 mM dRib and 100 μM DFO, 20 μM Fer‐1, or 20 μM Lip‐1 for 6 h. MDA levels were measured using the EZ‐Lipid Peroxidation Assay Kit (DoGenBio, South Korea), while 4‐HNE was measured using the Universal 4‐Hydroxynonenal ELISA Kit (Novus). The results are expressed as nmol/mg protein and ng/mg protein, respectively.

### 2.9 Assessment of Lipid ROS Levels

Intracellular lipid ROS levels were measured using flow cytometry with the dye, C11‐BODIPY (Molecular Probes). RIN5mF cells (1 × 10^6^ cells/well) were cultured in six‐well plates and stimulated with 30 mM dRib and 100 μM DFO, 20 μM Fer‐1, or 20 μM Lip‐1 for 6 h. The cells were treated with 4 μM C11‐BODIPY during the last 30 min, harvested, and resuspended in PBS. Lipid ROS levels were measured using a FACScan instrument (BD Biosciences). Data are expressed as the fold ratio of the mean fluorescence intensity relative to the control fluorescence value.

### 2.10 Measurement of Intracellular Adenosine Triphosphate Levels

Intracellular adenosine triphosphate (ATP) levels were determined using the Invitrogen ATP Determination Kit (Thermo Fisher Scientific). RIN5mF cells (1 × 10^6^ cells/well) were cultured in six‐well plates for 24 h, followed by stimulation with 200 μM H_2_O_2_ and 30 mM dRib for various times. After stimulation, the cells were lysed, and ATP levels were measured based on luminescence and normalized to the total protein concentration.

### 2.11 Transmission Electron Microscopy

To evaluate ultrastructural changes associated with ferroptosis, RIN5mF cells were cultured in 100 mm tissue culture dishes at a density of 1 × 10^6^ cells per dish and treated with 30 mM dRib for 6 h. Cells were fixed in 2% paraformaldehyde and 2.5% glutaraldehyde in 0.1 M phosphate buffer (pH 7.4) for 30 min at room temperature and washed three times with the same buffer. Post‐fixation was conducted using 1% osmium tetroxide (OsO_4_) in 0.1 M phosphate buffer for 90 min. After dehydration through a graded ethanol series, the cells were embedded in Epon‐812 resin (OKEN, Japan). Ultrathin sections (~70 nm) were prepared using an ultramicrotome (UC6, Leica, Germany), stained with 5% uranyl acetate followed by lead citrate, and examined using a transmission electron microscope (H‐7650; Hitachi, Tokyo, Japan) at an accelerating voltage of 80 kV.

### 2.12 RNA Isolation and Reverse Transcription‐Quantitative Polymerase Chain Reaction

Total RNA was extracted using TRIzol (Gibco Invitrogen), and reverse transcription was performed using MMLV reverse transcriptase (MGmed Corporation). Reverse transcription‐quantitative polymerase chain reaction (RT‐qPCR) was conducted using KAPA SYBR FAST qPCR Master Mix (KAPA Biosystems) with an iQTM 5 Multicolor Real‐Time PCR Detection system (Bio‐Rad, Hercules, CA, USA). Gene expression levels were normalized to β‐actin levels as a control. The sequences of all primers are presented in Table 1.

**TABLE 1:** Primer sequences used for RT‐qPCR.

Gene	Forward primer (5′–3′)	Reverse primer (5′–3′)
SLC7A11	GACAGTGTGTGCATCCCCTT	GCATGCATTTCTTGCACAGTTC
GPX4	CGCCGAGTGTGGTTTACGA	GCTCCTGCCTCCCGAACT
ACSL4	ATATTCGTCACCACTCACA	AACCTTGCTCATAACATTCTT
CHAC1	GCCCTGTGGATTTTCGGGTA	ATCTTGTCGCTGCCCCTATG
PTGS2	GGGAGTCTGGAACATTGTGAA	GTGCACATTGTAAGTAGGTGGACT
TRIM26	TCAGTGAGCTTGACCGGTTG	TTGGTGTCCTGTGTAGTGCAG
OTUB1	GCGACCACATCCACATCA	TAGGACCATTTACAACCACAGA
β‐actin	TCCTGGCCTCACTGTCCAC	GGGCCGGACTCATCGTACT

### 2.13 Western Blotting

Cells were lysed, and equal amounts of protein were separated by sodium dodecyl sulfate‐polyacrylamide gel electrophoresis, transferred to a nitrocellulose membrane, and blocked with BSA. The membranes were incubated with primary antibodies against specific proteins, followed by horseradish‐peroxidase‐conjugated secondary antibodies. Bands were visualized using Western Lightning Plus‐ECL (PerkinElmer).

### 2.14 Ubiquitination Assay and Co‐Immunoprecipitation

Immunoprecipitation was performed using Protein A/G Magnetic Beads (Thermo Fisher Scientific). Lysates from stimulated RIN5mF cells were incubated with magnetic‐bead‐antibody mixtures overnight. Immunoprecipitants were eluted and analyzed by western blotting using appropriate antibodies. HEK293T cells were co‐transfected with HA‐Ub, SLC7A11, and/or OTUB1, followed by dRib treatment. Immunoprecipitants were analyzed by western blotting using an anti‐SLC7A11 antibody. Co‐immunoprecipitation was performed similarly, followed by immunoblotting for specific protein interactions.

### 2.15 Statistical Analysis

Data are presented as the mean ± standard deviation. Group comparisons were performed using one‐way analysis of variance followed by Duncan's post hoc test, with a *p*‐value < 0.05 considered statistically significant. Analyses were conducted using SPSS software (version 14.0; SPSS Inc.).

## 3 Results

### 3.1 2‐ME, an Iron Chelator, and Lipid Radical Scavengers Prevented dRib‐Induced Lipid Peroxidation and Cell Death

**FIGURE 1:**
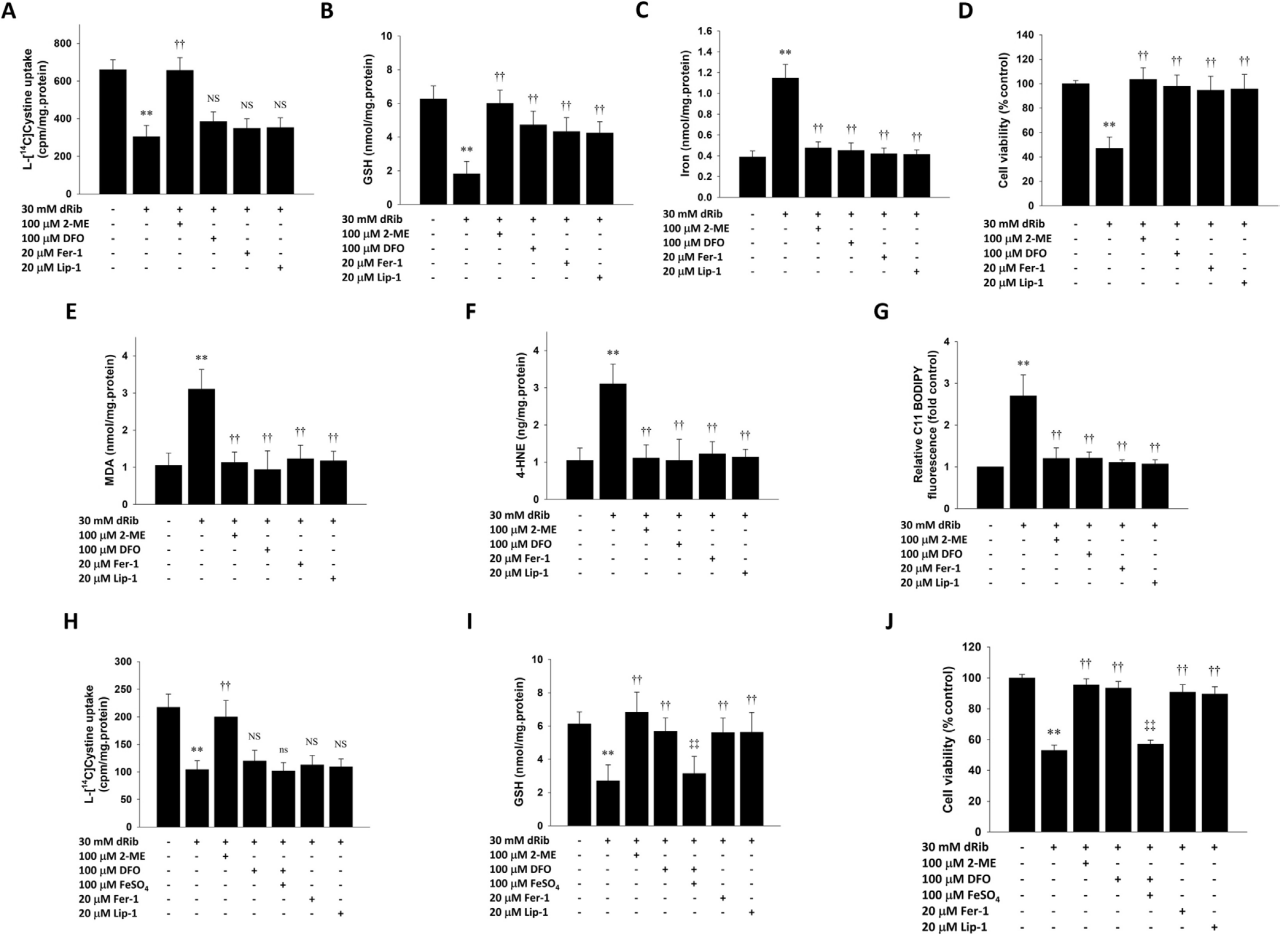
Protective effects of anti‐ferroptotic agents against dRib‐induced changes in RINm5F cells (A–G) and isolated islets (H–J). Cells were co‐treated with dRib and 2‐ME, DFO (±FeSO_4_), Fer‐1, or Lip‐1. [^14^C]cystine uptake (A, H), intracellular GSH (B, I), iron (C), MDA (E), 4‐HNE (F) levels, and cell viability (D, J), and lipid ROS levels (G) were assessed. This experiment was performed thrice, in triplicate. **p < 0.01 versus control; ††*p* < 0.01 versus dRib alone; NS & ns, no significant difference from the dRib‐alone & DFO plus dRib group, respectively.

In RINm5F cells, 2‐ME, which bypasses system χc^−^ to enhance cystine uptake, restored the decreased intracellular cystine uptake, reduced the increased intracellular iron levels, and decreased cell death caused by dRib. This result is consistent with our previous findings in RTECs [17]. In addition, 2‐ME restored the intracellular GSH content reduced by dRib to the control level. As expected, the iron chelator DFO and the lipophilic antioxidants Fer‐1 and Lip‐1 did not restore cystine uptake, but significantly restored intracellular GSH and iron levels and cell death (Figure 1A–D). dRib significantly increased intracellular levels of MDA and 4‐HNE, which were decreased by 2‐ME, DFO, Fer‐1, and Lip‐1 (Figure 1E,F). Flow cytometry also confirmed that lipid ROS levels increased due to dRib, and this was prevented by 2‐ME, DFO, Fer‐1, and Lip‐1 (Figure 1G and Figure S1). In isolated islets, dRib decreased cystine uptake, depleted GSH, and induced cell death. 2‐ME prevented the decrease in cystine uptake, GSH depletion, and cell death caused by dRib. DFO, Fer‐1, and Lip‐1 did not affect cystine uptake but did prevent GSH depletion and cell death induced by dRib. The protective effect of DFO was counteracted by the addition of FeSO_4_ (Figure 1H–J).

### 3.2 dRib Induced Morphological Changes in Mitochondria and Distinct Cell Death

**FIGURE 2:**
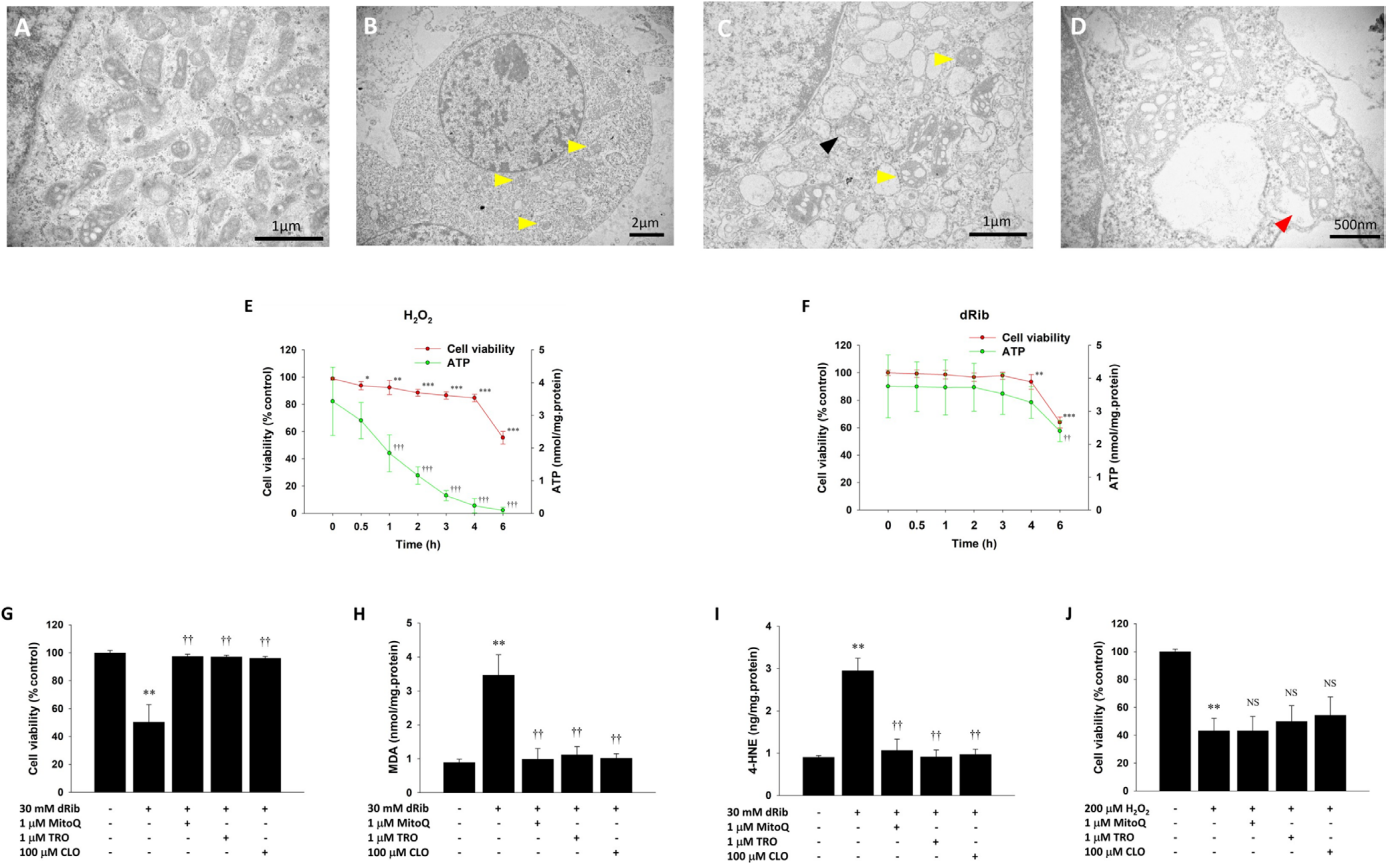
Characterization of dRib‐induced ferroptosis in RINm5F cells. (A–D) Cells were examined by TEM. Panel A shows mitochondria in control cells; panels B–D show dRib‐treated cells with yellow arrowheads indicating shrunken mitochondria, black arrowheads indicating reduced or absent cristae, and red arrowheads indicating outer membrane rupture. (E, F) Cells were exposed to 200 μM H_2_O_2_ or 30 mM dRib for 24 h, and ATP levels and viability were assessed by luminescence and LDH release assays, respectively. *p < 0.05, **p < 0.01, and ***p < 0.001 versus control in cell viability; ††*p* < 0.01 and †††*p* < 0.001 versus control in ATP levels. (G–J) Cells were co‐treated with 1 μM MitoQ, 1 μM TRO, or 100 μM CLO and either 30 mM dRib or 200 μM H_2_O_2_ for 6 h; cytotoxicity and lipid peroxidation (MDA, 4‐HNE) were measured using assay kits. **p < 0.01 versus control; ††*p* < 0.01 versus 30 mM dRib‐alone group, as determined by one way analysis of variance and Duncan's post hoc test. NS, no significant difference from 200 μM H_2_O_2_‐alone group. These experiments were performed thrice, in triplicate.

Ferroptosis is morphologically distinct from other forms of cell death. Upon dRib stimulation for 6 h, TEM observations of RINm5F cells revealed well‐preserved nuclear integrity and plasma membrane, without chromatin condensation or plasma membrane blebbing. However, characteristic mitochondrial changes were observed, including increased electron density, shrinkage, and round shape, along with reduced cristae and outer membrane rupture (Figure 2B–D). Ferroptosis is an energy‐requiring regulated cell death process. Unlike H_2_O_2_‐induced necrosis, in which intracellular ATP levels decrease from the beginning of the process, dRib‐treated RINm5F cells maintained their ATP levels for the first 4 h, before they decreased at 6 h, which coincided with the onset of decreased cell viability (Figure 2E,F). Additionally, inhibitors known to suppress ferroptosis—troglitazone, mitoquinone, and clorgyline—significantly prevented dRib‐induced lipid peroxidation and cell death but did not affect H_2_O_2_‐induced cell death (Figure 2G–J).

### 3.3 dRib Altered the Expression of Ferroptosis‐Related Molecules and SLC7A11 Overexpression Suppressed dRib‐Induced Lipid Peroxidation

**FIGURE 3:**
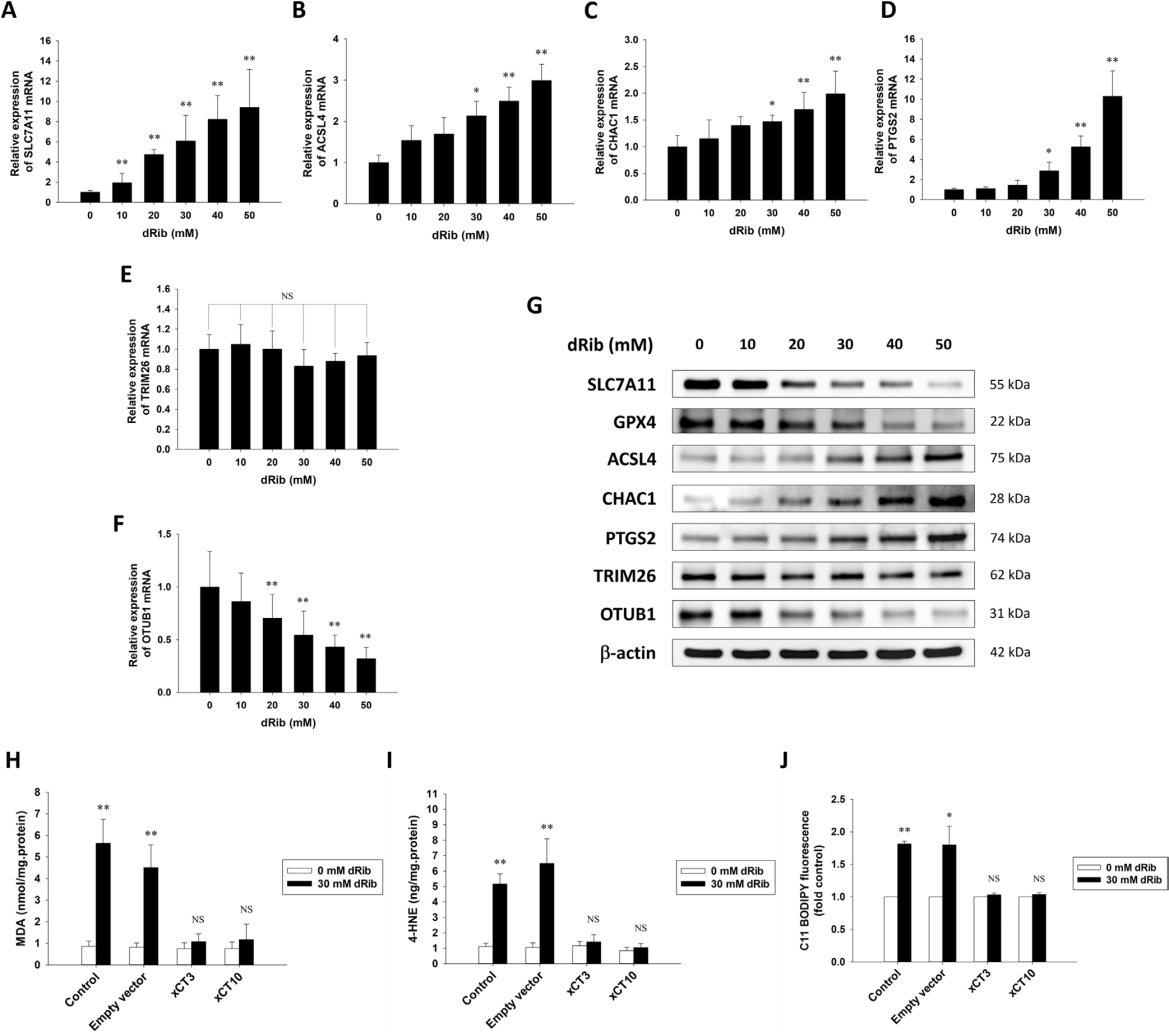
Molecular changes induced by dRib and protective effects of SLC7A11 overexpression in RIN5mF cells. (A–F) Cells were treated with 0–50 mM dRib for 4 h in RPMI‐1640 with 10% FBS. mRNA expression of ferroptosis‐related genes was quantified by qRT‐PCR. (G) Protein levels of SLC7A11, GPX4, ACSL4, CHAC1, PTGS2, TRIM26, and OTUB1 were analyzed by western blotting; β‐Actin was used as a loading control. (H–J) To assess the effects of SLC7A11 overexpression, xCT‐transduced and control cells were treated with 30 mM dRib for 6 h. Lipid peroxidation (MDA, 4‐HNE) was measured using assay kits (H, I), and lipid ROS levels were assessed by flow cytometry using C11‐BODIPY (J). Experiments were performed in triplicate. *p < 0.05 and **p < 0.01 versus 0 mM dRib; NS, no significant difference from 0 mM dRib.

dRib dose‐dependently increased the expression levels of well‐known ferroptosis markers, acyl‐CoA synthetase long‐chain family member 4 (ACSL4), ChaC glutathione specific gamma‐glutamylcyclotransferase 1 (CHAC1), and prostaglandin‐endoperoxide synthase 2 (PTGS2), at both the mRNA and protein levels. In addition, GPX4 protein expression, another crucial regulator of ferroptosis, decreased in a dose‐dependent manner. Notably, while *SLC7A11* mRNA levels increased in a dose‐dependent manner, its protein levels decreased in a dose‐dependent manner (Figure 3A–G). Similar trends were observed in isolated islets, in which dRib increased the expression levels of *SLC7A11*, *ACSL4*, *CHAC1*, and *PTGS2* mRNA (Figure S2A–D). To confirm that dRib‐induced ferroptosis is due to decreased SLC7A11 expression levels, we overexpressed SLC7A11 in RINm5F cells using a lentivirus vector. SLC7A11 overexpression significantly suppressed the dRib‐induced increases in intracellular MDA, 4‐HNE, and lipid ROS levels (Figure 3H–J and Figure S3). These results suggest that dRib induces lipid peroxidation and cell death by reducing cystine uptake through the system χc^−^.

### 3.4 dRib Induced SLC7A11 Protein Degradation via the Ubiquitin‐Proteasome System and Reduced OTUB1 Expression Levels

**FIGURE 4:**
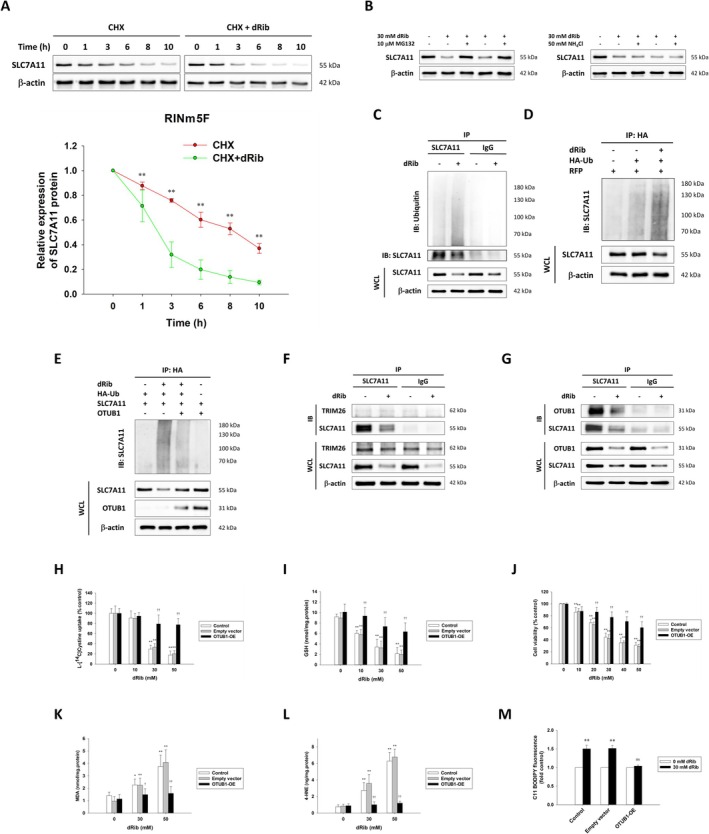
Investigating SLC7A11 ubiquitination and the role of OTUB1 in ferroptosis regulation. (A) RIN5mF cells were treated with 40 μg/mL CHX in the presence or absence of 30 mM dRib, and SLC7A11 protein stability was assessed by immunoblotting and quantified over time. **p < 0.01 versus CHX plus dRib group. (B) Cells were treated with 30 mM dRib with or without MG132 or NH_4_Cl to determine whether SLC7A11 degradation was mediated via the proteasome or lysosome. (C–E) SLC7A11 ubiquitination was analyzed by immunoprecipitation/immunoblotting in RIN5mF (C) or HEK293T cells co‐transfected with HA‐Ub, SLC7A11, and/or OTUB1 and treated with or without dRib (D, E). (F, G) Interaction of SLC7A11 with TRIM26 and OTUB1 was evaluated by co‐immunoprecipitation in dRib‐treated RIN5mF cells. (H–M) Effects of OTUB1 overexpression on dRib‐induced changes in [^14^C]cystine uptake (H), GSH content (I), cell viability (J), lipid peroxidation (K, L), and lipid ROS (M) were assessed using standard assays. Experiments were performed in triplicate (H–L) or quadruplicate (M). *p < 0.05 and **p < 0.01 versus 0 mM dRib group; †*p* < 0.05 and ††*p* < 0.01 versus control and empty vector groups; ns, no significant difference from 0 mM dRib.

Although dRib increased *SLC7A11* mRNA levels, it decreased SLC7A11 protein expression levels. This reduction in SLC7A11 protein levels was accelerated, even in the presence of the protein biosynthesis inhibitor cycloheximide, suggesting that dRib induced the post‐translational degradation of SLC7A11 (Figure 4A). Further investigation revealed that the proteasome inhibitor MG132 restored SLC7A11 protein levels reduced by dRib, whereas the lysosomal inhibitor NH_4_Cl did not, indicating proteasomal degradation (Figure 4B). Immunoprecipitation and immunoblotting confirmed the increased ubiquitination of SLC7A11 in dRib‐treated cells (Figure 4C,D). In addition, dRib did not affect the expression of TRIM26, an E3 ubiquitin ligase, but dose‐dependently decreased the mRNA and protein expression levels of OTUB1, a deubiquitinating enzyme (DUB) in RIN5mF cells (Figure 3E–G). Similar results were observed in rat islets (Figure S2E–F).

### 3.5 OTUB1 Interacted With SLC7A11, Inhibited Its Ubiquitination, and Prevented dRib‐Induced Ferroptosis

Co‐immunoprecipitation assays confirmed that SLC7A11 interacted with OTUB1, but not with TRIM26 (Figure 4F,G). Ubiquitination assays in HEK‐293 T cells showed that OTUB1 overexpression reduced dRib‐induced SLC7A11 ubiquitination (Figure 4E), suggesting that OTUB1 prevented SLC7A11 degradation by inhibiting its ubiquitination. To confirm that a reduction in OTUB1 levels is the mechanism responsible for dRib‐induced ferroptosis, we overexpressed OTUB1 in RINm5F cells. CHX chase assays revealed that dRib accelerated the degradation of SLC7A11 protein in control cells (Figure 4A), whereas this effect was abolished in OTUB1‐overexpressing cells (Figure S4). Moreover, OTUB1 overexpression significantly restored intracellular cystine uptake, GSH content, and cell viability, and reduced lipid peroxidation product and intracellular ROS levels in dRib‐treated cells (Figure 4H–M, Figure S5). These findings indicate that OTUB1 stabilizes SLC7A11 by reducing its ubiquitination and thereby prevents dRib‐induced ferroptosis through maintaining cystine transport via system χc‐.

## 4 Discussion

In this study, we found that, in pancreatic β‐cells, dRib induced ferroptosis by depleting intracellular GSH through reduced cystine transport. This mechanism involved the increased ubiquitination and proteasomal degradation of the SLC7A11 protein, the functional subunit of system χc‐, due to decreased expression levels of OTUB1, a type of DUB. Understanding the ferroptosis pathways in β‐cells, which are vulnerable to oxidative damage, will contribute to clarifying the pathogenesis of diabetes and developing new treatment strategies.

β‐Cell death induced by dRib exhibited all major characteristics of ferroptosis. Specifically, increases in intracellular iron, MDA, 4‐HNE, and lipid ROS were observed, all of which were inhibited by iron chelators and lipophilic antioxidants. Mitochondrial shrinkage was confirmed by TEM, and changes in ferroptosis‐associated molecules were also detected, including regulators such as ACSL4 and markers such as PTGS2 and CHAC1. Notably, the upregulation of CHAC1 is a feature specific to ferroptosis induced by system χc^−^ inhibition [18]. 2‐ME, which is known to bypass system χc^−^ and enhance cystine uptake [19], restored the reduced levels of cystine and GSH caused by dRib and suppressed ferroptosis. In contrast, DFO, Fer‐1, and Lip‐1 inhibited ferroptosis but did not affect cystine transport. In our previous studies, we showed that overexpression of SLC7A11 reversed the dRib‐induced reductions in cystine uptake, GSH levels, and cell viability [15, 17]. In the present study, SLC7A11 overexpression also suppressed the increases in lipid ROS and lipid peroxidation products in β‐cells. These findings demonstrate that dRib induces β‐cell ferroptosis by inhibiting cystine transport via system χc^−^, leading to GSH depletion and oxidative lipid damage.

Since necrosis is an accidental cell death, ATP is depleted from the early stages of cell death [20]. In contrast, in ferroptosis, which is a form of regulated cell death, intracellular ATP is maintained until just before cell death [21]. In our study, dRib‐treated cells retained ATP levels until death, unlike H_2_O_2_‐treated cells, which showed early ATP depletion. This indicates that dRib‐induced death is an active, regulated process, consistent with ferroptosis. Since iron chelators and lipid radical scavengers also inhibit H_2_O_2_‐induced cytotoxicity, their effects alone do not confirm ferroptosis [22]. However, H_2_O_2_‐induced cell death, characterized by early ATP loss, cannot be defined as ferroptosis. To further validate the ferroptotic nature of dRib‐induced death, we tested additional ferroptosis inhibitors. Troglitazone, an ACSL4 inhibitor [23], along with MitoQ and clorgyline—agents that reduce mitochondrial ROS [24]—effectively suppressed dRib‐induced lipid peroxidation and cell death, but had no effect on H_2_O_2_‐induced death. These results, combined with dRib's ATP‐preserving profile and specific sensitivity to ferroptosis inhibitors, confirm that dRib induces ferroptosis rather than nonspecific oxidative damage.

To clarify how dRib promotes SLC7A11 degradation via the ubiquitin‐proteasome system (UPS), we focused on two key enzymes: TRIM26 and OTUB1. The reason for this focus is that SLC7A11 is regulated at various levels, including transcriptional, posttranscriptional, translational, and posttranslational. Since our CHX chase assay showed that dRib shortened the half‐life of the SLC7A11 protein, we determined that SLC7A11 was affected at the posttranslational level. Furthermore, treatment with MG132 prevented the reduction in protein expression, and immunoprecipitation confirmed an increase in SLC7A11 ubiquitination, indicating that the mechanism of dRib involves UPS‐mediated degradation of the SLC7A11 protein. To our knowledge, the molecules known to regulate UPS‐mediated degradation of SLC7A11 are TRIM26 and OTUB1. TRIM26, an E3 ligase, enhances SLC7A11 ubiquitination and induces ferroptosis [25], while OTUB1, a DUB, stabilizes SLC7A11 and suppresses ferroptosis [26, 27]. Our study demonstrated that dRib suppresses OTUB1 expression in β‐cells, thereby promoting SLC7A11 degradation and inducing ferroptosis. This is the first report implicating the OTUB1‐SLC7A11 axis in β‐cell ferroptosis. Given that both OTUB1 and SLC7A11 are upregulated in various cancers [3, 26], dRib's action on this pathway suggests its potential as an anticancer agent.

This study has several limitations. First, we did not assess β‐cell function (e.g., insulin secretion), as our focus was on cell death. Given that β‐cell mass—primarily determined by β‐cell death—is a major contributor to the development of diabetes [28], this does not undermine the study's significance. Second, experiments were limited to cell lines and isolated rat islets; in vivo validation is needed. Third, while we found that dRib decreases OTUB1 expression, leading to increased SLC7A11 ubiquitination and ferroptosis, the exact mechanism—whether via canonical DUB activity or non‐canonical inhibition of E2 enzymes [29]—remains unclear and warrants further investigation. Fourth, Liu et al. [26] reported that OTUB1 knockout alone did not induce cell death unless additional stimuli such as tert‐butyl hydroxide, erastin, or cystine starvation were applied. This suggests that OTUB1 deficiency by itself may not be sufficient to trigger cell death, and that dRib‐induced ferroptosis may involve additional mechanisms beyond OTUB1 downregulation, warranting further investigation.

In conclusion, this study demonstrated that dRib induces ferroptosis in pancreatic β‐cells by downregulating OTUB1, thereby promoting SLC7A11 degradation and impairing cystine uptake and GSH synthesis. Overexpression of OTUB1 or SLC7A11 alleviated lipid peroxidation and cell death, highlighting their protective roles. These findings suggest that targeting the OTUB1–SLC7A11 axis may preserve β‐cell survival and offer a potential therapeutic strategy for diabetes and possibly cancer.

## Author Contributions

Gwanpyo Koh conceived and designed the study. Ju Young Bae, Soyeon Yoo, and Eui Tae Kim performed the experiments. Gwanpyo Koh, Soyeon Yoo, Ju Young Bae, and Miyeon Kim analyzed and interpreted data. Gwanpyo Koh, Soyeon Yoo, Dongkyu Kim, and Miyeon Kim wrote the manuscript. Eui Tae Kim reviewed and edited the manuscript. Gwanpyo Koh, Soyeon Yoo, Dongkyu Kim, and Sang Ah Lee acquired funding. Gwanpyo Koh, Soyeon Yoo, and Ju Young Bae confirm the authenticity of all the raw data. All authors read and approved the final version of the manuscript.

## Conflicts of Interest

The authors declare no conflicts of interest.

## Supporting information


**Figure S1:** Effects of 2‐ME (A), DFO (B), Fer‐1 (C), and Lip‐1 (D) on dRib‐induced lipid ROS elevation in RINm5F cells. Cells were co‐treated with dRib and each agent, and lipid ROS levels were measured by flow cytometry using C11‐BODIPY. Representative histograms from four independent experiments are shown.

**Figure S2:** Expression of SLC7A11 (A), ACSL4 (B), CHAC1 (C), PTGS2 (D), TRIM26 (E), and OTUB1 (F) mRNA in isolated islets after treatment with various concentrations of 2‐deoxy‐d‐ribose (dRib). Islets were exposed to 0–30 mM dRib for 6 h in RPMI‐1640 with 10% FBS. mRNA levels were quantified by qRT‐PCR using the 2^−ΔΔCT^ method. This experiment was performed thrice, in triplicate. *p < 0.05 and **p < 0.01, versus 0 mM dRib group; NS, no significant difference from 0 mM dRib group.

**Figure S3:** Effects of SLC7A11 overexpression on dRib‐induced lipid ROS in RIN5mF cells. Cells were treated with 30 mM dRib for 6 h in RPMI‐1640 with 10% FBS. Lipid ROS levels were measured by flow cytometry after staining with 4 μM C11‐BODIPY for 30 min. Shown are representative histograms from four independent experiments comparing control, empty vector, and two xCT‐overexpressing clones (xCT3 and xCT10).

**Figure S4:** Effects of OTUB1 overexpression on SLC7A11 protein stability under dRib + CHX treatment. RIN5mF cells stably overexpressing OTUB1 were treated with 40 μg/mL cycloheximide (CHX) in the presence or absence of 30 mM dRib for the indicated times. SLC7A11 protein levels were analyzed by immunoblotting and quantified relative to β‐actin. Data are presented as mean ± SD of three independent experiments. ns: no significant difference from versus CHX plus dRib group.

**Figure S5:** Effects of OTUB1 overexpression on dRib‐induced lipid ROS in RIN5mF cells. Cells were treated with 0 or 30 mM dRib for 6 h in RPMI‐1640 medium with 10% FBS. Lipid ROS levels were measured by flow cytometry after staining with 4 μM C11‐BODIPY for 30 min. Shown are representative histograms from four independent experiments comparing control, empty vector, and OTUB1‐overexpressing clones.

## Data Availability

The data used to support the findings of this study are available from the corresponding author upon request.
