# Supplementary figures and images for: OTUB1 Modulates Ferroptosis by Regulating SLC7A11 Ubiquitination in Pancreatic β‐Cells
Source: FASEB J. 2025 Oct 12;39(20):e71128. doi: 10.1096/fj.202502289R (PMC12516175; doi:10.1096/fj.202502289R)

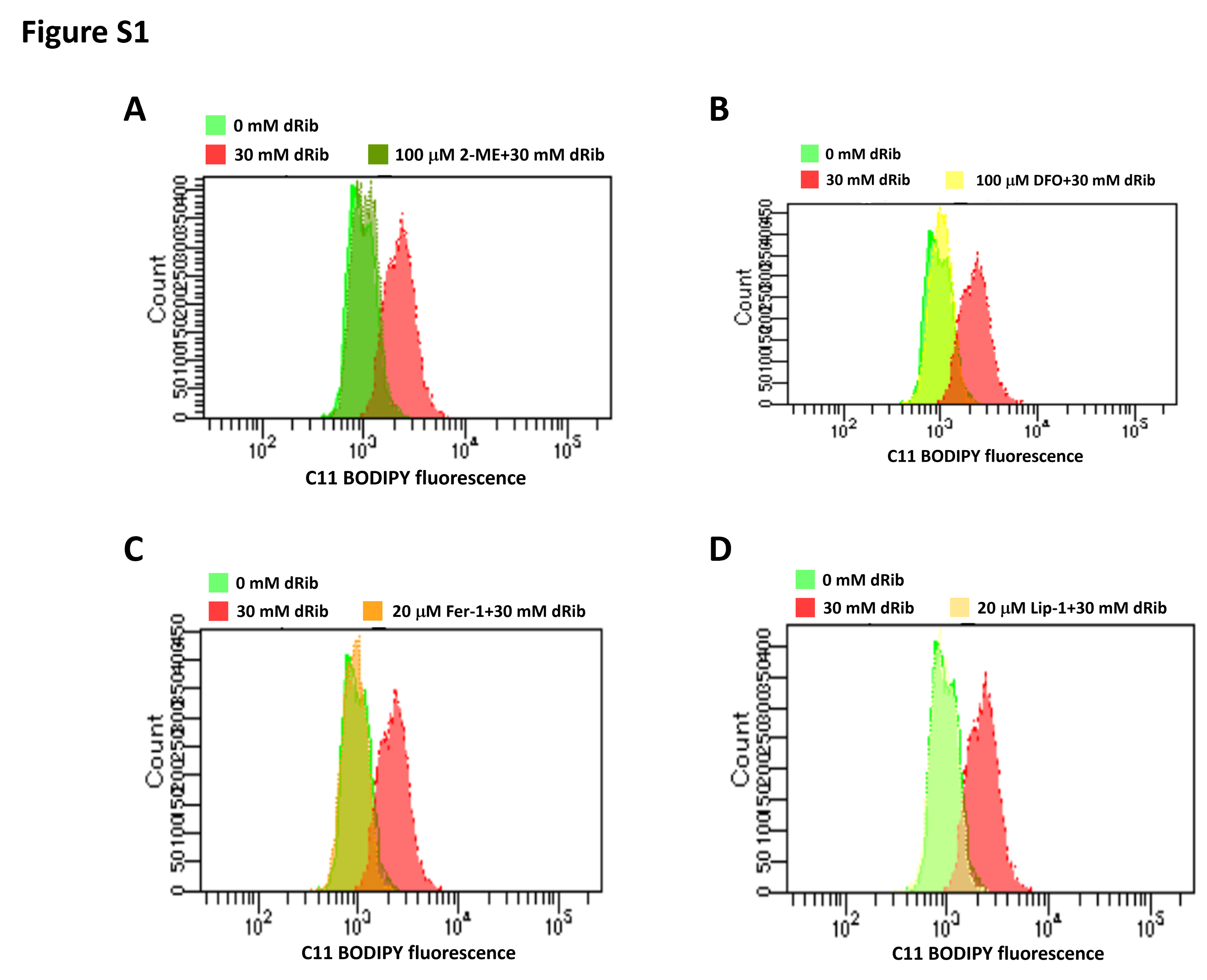

Supplement: Supplementary file 1 — Figure S1: Effects of 2‐ME (A), DFO (B), Fer‐1 (C), and Lip‐1 (D) on dRib‐induced lipid ROS elevation in RINm5F cells. Cells were co‐treated with dRib and each agent, and lipid ROS levels were measured by flow cytometry using C11‐BODIPY. Representative histograms from four independent experiments are shown. [file FSB2-39-e71128-s001.tif]

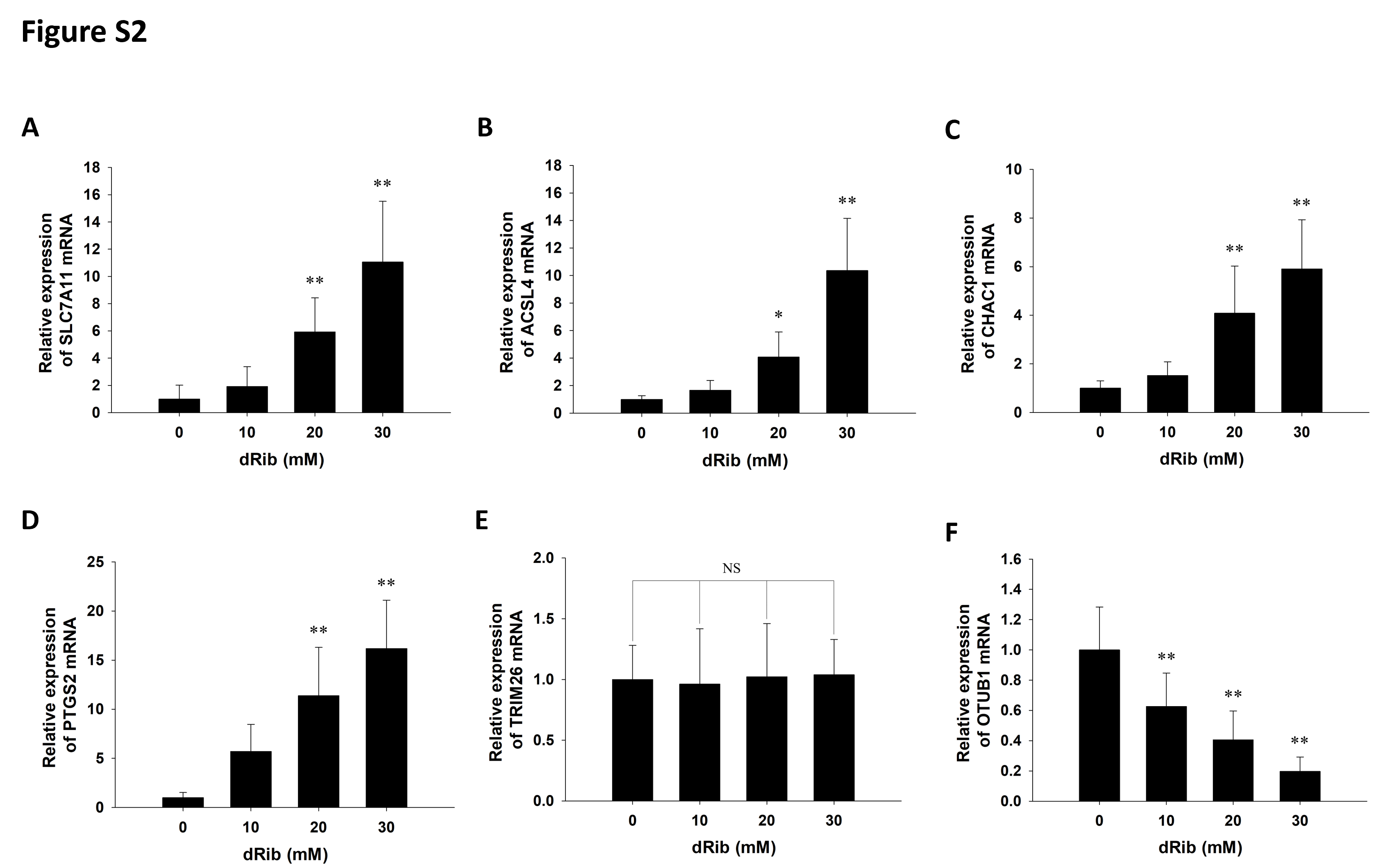

Supplement: Supplementary file 2 — Figure S2: Expression of SLC7A11 (A), ACSL4 (B), CHAC1 (C), PTGS2 (D), TRIM26 (E), and OTUB1 (F) mRNA in isolated islets after treatment with various concentrations of 2‐deoxy‐d‐ribose (dRib). Islets were exposed to 0–30 mM dRib for 6 h in RPMI‐1640 with 10% FBS. mRNA levels were quantified by qRT‐PCR using the 2−ΔΔCT method. This experiment was performed thrice, in triplicate. *p < 0.05 and **p < 0.01, versus 0 mM dRib group; NS, no significant difference from 0 mM dRib group. [file FSB2-39-e71128-s005.tif]

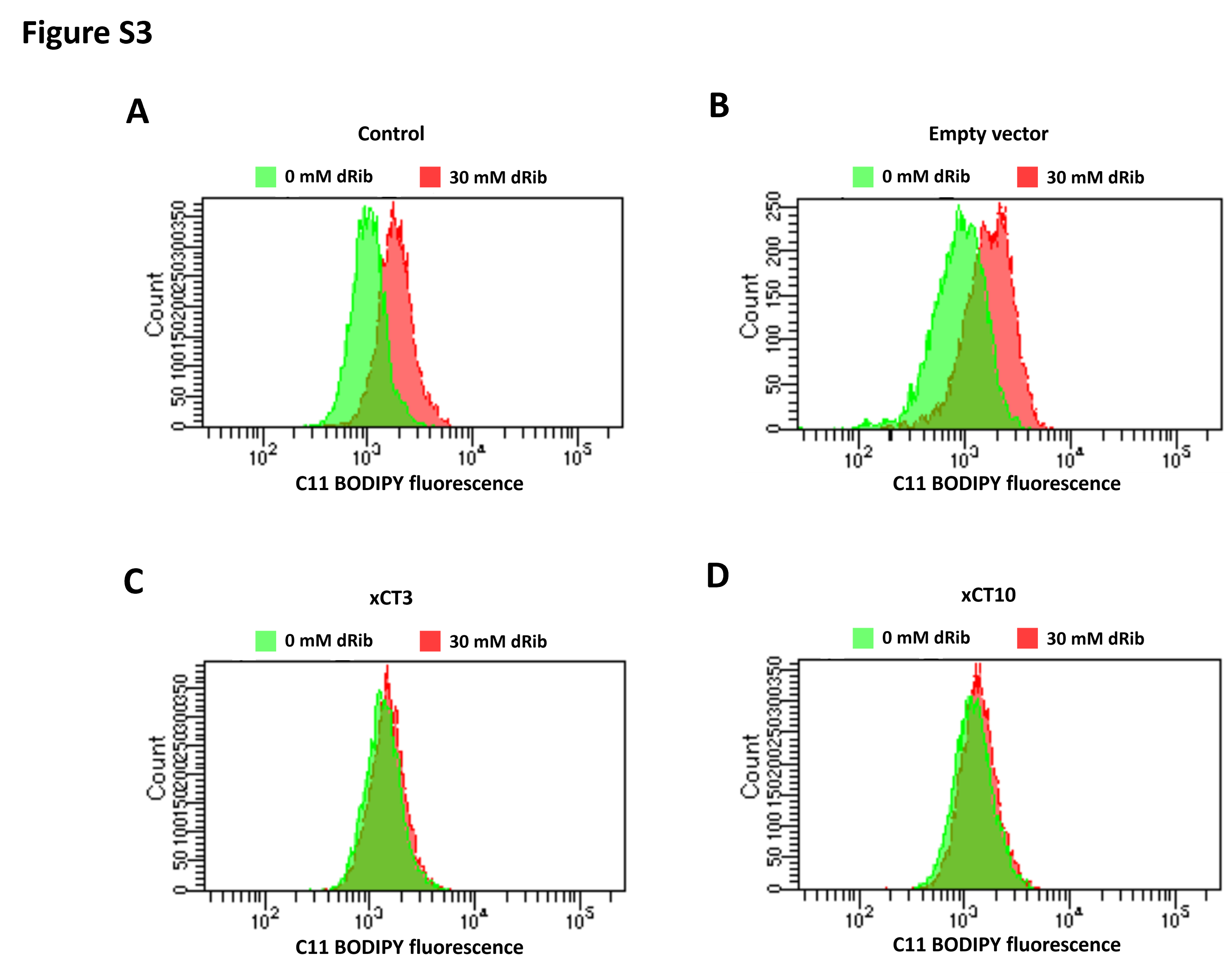

Supplement: Supplementary file 3 — Figure S3: Effects of SLC7A11 overexpression on dRib‐induced lipid ROS in RIN5mF cells. Cells were treated with 30 mM dRib for 6 h in RPMI‐1640 with 10% FBS. Lipid ROS levels were measured by flow cytometry after staining with 4 μM C11‐BODIPY for 30 min. Shown are representative histograms from four independent experiments comparing control, empty vector, and two xCT‐overexpressing clones (xCT3 and xCT10). [file FSB2-39-e71128-s004.tif]

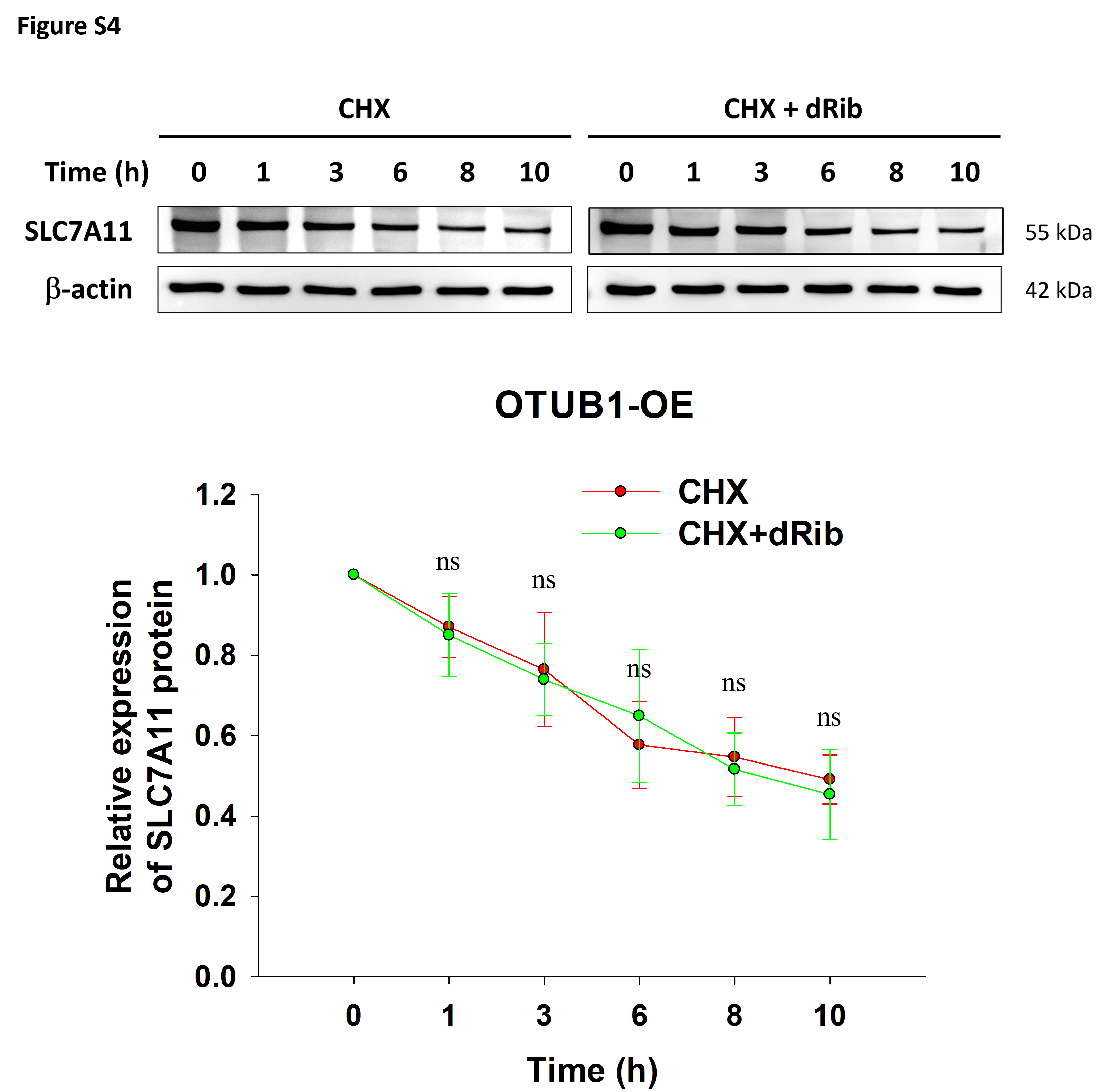

Supplement: Supplementary file 4 — Figure S4: Effects of OTUB1 overexpression on SLC7A11 protein stability under dRib + CHX treatment. RIN5mF cells stably overexpressing OTUB1 were treated with 40 μg/mL cycloheximide (CHX) in the presence or absence of 30 mM dRib for the indicated times. SLC7A11 protein levels were analyzed by immunoblotting and quantified relative to β‐actin. Data are presented as mean ± SD of three independent experiments. ns: no significant difference from versus CHX plus dRib group. [file FSB2-39-e71128-s002.tif]

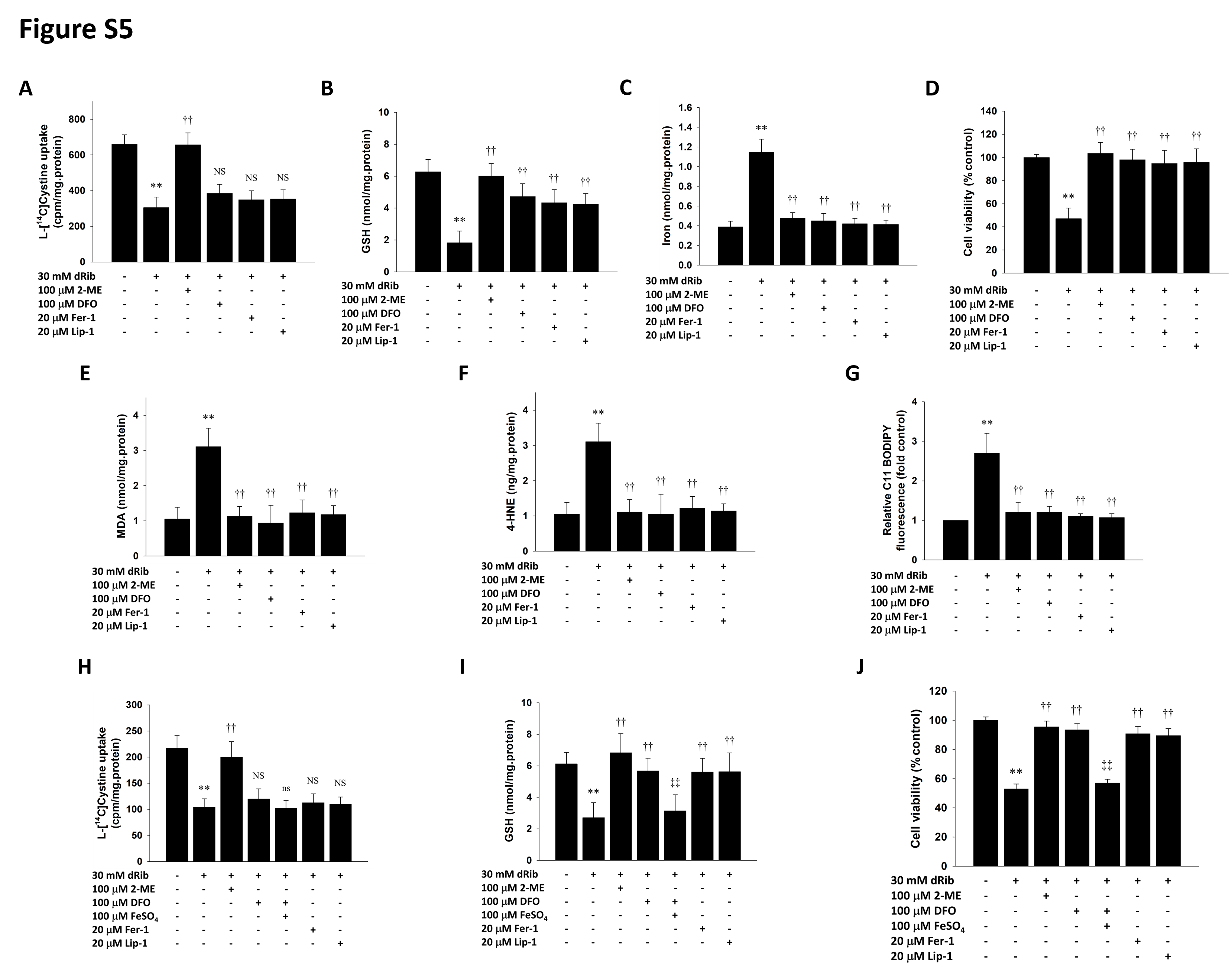

Supplement: Supplementary file 5 — Figure S5: Effects of OTUB1 overexpression on dRib‐induced lipid ROS in RIN5mF cells. Cells were treated with 0 or 30 mM dRib for 6 h in RPMI‐1640 medium with 10% FBS. Lipid ROS levels were measured by flow cytometry after staining with 4 μM C11‐BODIPY for 30 min. Shown are representative histograms from four independent experiments comparing control, empty vector, and OTUB1‐overexpressing clones. [file FSB2-39-e71128-s003.tif]
